# The nature and prevalence of chronic pain in homeless persons: an observational study

**DOI:** 10.12688/f1000research.2-164.v1

**Published:** 2013-07-30

**Authors:** Rebecca Fisher, Judith Ewing, Alice Garrett, E Katherine Harrison, Kimberly KT Lwin, Daniel W Wheeler

**Affiliations:** 1 Department of Primary Health Care Sciences, University of Oxford, Radcliffe Observatory Quarter, Woodstock Road, Oxford, OX2 6GG, UK; 2 Public Health Agency, 12-22 Linenhall Street, Belfast, BT2 8BS, UK; 3 University of Cambridge School of Clinical Medicine, Addenbrooke's Hospital, Box 111, Hills Road, Cambridge, CB2 0SP, UK; 4 Division of Anaesthesia, University of Cambridge Addenbrooke's Hospital, Box 93, Hills Road, Cambridge, CB2 2QQ, UK

## Abstract

**Background: **Homeless people are known to suffer disproportionately with health problems that reduce physical functioning and quality of life, and shorten life expectancy. They suffer from a wide range of diseases that are known to be painful, but little information is available about the nature and prevalence of chronic pain in this vulnerable group. This study aimed to estimate the prevalence of chronic pain among homeless people, and to examine its location, effect on activities of daily living, and relationship with alcohol and drugs.

**Methods:** We conducted face-to-face interviews with users of homeless shelters in four major cities in the United Kingdom, in the winters of 2009-11. Participants completed the Brief Pain Inventory, Short Form McGill Pain questionnaire, Leeds Assessment of Neuropathic Symptoms and Signs, and detailed their intake of prescribed and unprescribed medications and alcohol. We also recorded each participant’s reasons for homelessness, and whether they slept rough or in shelters.

**Findings:** Of 168 shelter users approached, 150 (89.3%) participated: 93 participants (63%) reported experiencing pain lasting longer than three months; the mean duration of pain experienced was 82.2 months. The lower limbs were most frequently affected. Opioids appeared to afford a degree of analgesia for some, but whilst many reported symptoms suggestive of neuropathic pain, very few were taking anti-neuropathic drugs.

**Interpretation:** The prevalence of chronic pain in the homeless appears to be substantially higher than the general population, is poorly controlled, and adversely affects general activity, walking and sleeping. It is hard to discern whether chronic pain is a cause or effect of homelessness, or both. Pain is a symptom, but in this challenging group it might not always be possible to treat the underlying cause. Exploring the diagnosis and treatment of neuropathic pain may offer a means of improving the quality of these vulnerable people’s lives.

## Introduction

Homeless people suffer disproportionately from health problems, to such an extent that the life expectancy of a person sleeping on the streets is 42–52 years ^1– 4^. Their lifestyle predisposes them to various potentially painful and unpleasant complaints (including dental caries, trench foot, infectious diseases, peripheral neuropathy and depression), but such conditions often go untreated due to multiple barriers to care ^5– 8^. Inadequate living conditions, frequent exposure to the elements, and violence, substance misuse, and poor nutrition combine with inadequate access to healthcare to exacerbate health problems ^4, 9– 11^.

In England, the Housing Act 1996 defines a person as being homeless if there is no accommodation that they are entitled to occupy; or they have accommodation but it is not reasonable for them to continue to occupy it ^12^. Although the United Kingdom (UK) government has reported an overall annual decline in homelessness as defined by this legislation since 2004, the most recent data show that this trend has reversed. In the second quarter of 2012, local authorities in England accepted 12,960 applicants as being homeless, and Rough Sleeping England estimated that 1,768 people were ‘sleeping rough’ on the streets ^13, 14^. As government figures do not include people who satisfy the legal definition of homelessness but who have not applied to be classified as such, it is likely that true incidence of homelessness is substantially higher. Crisis, a charity representing the interests of single homeless people in the UK, estimates that there are around 400,000 ‘hidden homeless’ in England, Wales and Scotland ^3^.

Large epidemiological studies in Australia, Europe and Scotland, using a definition of chronic pain as ‘pain that persists beyond normal tissue healing time, which is assumed to be three months’ ^15^ have found a prevalence ranging from 17% to 50% in the general population ^16– 18^. Given the increased prevalence of health problems in homeless people ^4– 8^, we hypothesised that they may also experience more pain than the general population.

To date there have been no studies primarily addressing the prevalence of pain in homeless persons, although in a study from Canada, almost 52% of randomly selected homeless individuals fulfilled the criteria for chronic pain ^19^. Pain has featured as a secondary topic in studies of oral health, end of life care, and the measurement of pain in the context of homelessness ^5, 20, 21^. Pain is a symptom, and, although it would be preferable to treat the underlying causes, barriers to care and the presence of potentially irreversible disease mean that it is important to gain insights into the quantity and character of pain that homeless people experience, its impact on function, and the strategies they use to manage it. This study addresses pain as a primary focus, as better characterisation of pain may pave the way for targeted intervention.

We used standard questionnaires to estimate the prevalence, duration and nature of pain in homeless people, and examine its character, location, severity, impact on activities of daily living, and the strategies used for pain relief.

## Methods

We conducted a pilot questionnaire survey of users at two homeless shelters in Cambridge, UK, over 3 months in late 2009 and early 2010. Five of the authors acted as interviewers (RFRF, JCE, AG, EKH and KKTL), having received training in the use of the Brief Pain Inventory (BPI) and short form McGill (SF-MPQ) questionnaires from DWW ^22– 25^. Introductions to shelter users were facilitated by shelter staff when required. Written consent was obtained from all participants.

Participants in our pilot study completed the BPI and the SF-MPQ questionnaires face-to-face during a single interview. Interviews followed a standardised format, with questions read aloud to avoid issues with literacy. Participants reporting pain for more than 3 months were asked to rate its average and its greatest intensities during the past 24 hours on an 11-point numeric rating scale (NRS 0–10, where 0 represents no pain and 10 equates to the worst pain imaginable), to mark primary pain location on whole-body diagrams, and provide a list of their current prescribed and unprescribed medications. Each participant was asked to detail their alcohol use in the past 24 hours (converted into number of units), and their recreational drug use (by drug used and amount).

Having collected and analysed these pilot data, we extended the study to include more centres, recruiting homeless adults from five shelters in Oxford, Belfast, and London. We also recruited from day centres that aim to help homeless people ‘sleeping rough’ on the streets. In total, eight institutions were approached, but one declined to participate as they dealt mostly with homeless adolescents in whom they did not consider pain to be a problem. Participants from these additional centres were asked further questions concerning the main reason for their homelessness if possible, which was categorised into drug or alcohol misuse, mental health problems, family or relationship breakdown, financial difficulties, and leaving prison or the armed forces. These participants were also asked their duration of pain, how long they had been homeless, whether they predominantly slept rough on the streets, or under a roof in night shelters or hostels, for example.

As our pilot data suggested a high prevalence of neuropathic pain, these participants were also asked to complete the Leeds Assessment of Neuropathic Symptoms and Signs questionnaire and examination (LANSS) ^26^, to improve the precision with we could detect the prevalence of neuropathic pain ^27^.

Using the BPI, short form McGill and LANSS questionnaires together provides a comprehensive analysis of an individual’s pain that we thought would be easy to use and appropriate for the homeless population; cover current treatments for pain and their perceived efficacy rated on a percentage scale of pain relief and assess the extent to which pain interferes with daily functions ^28^.

### Inclusion and exclusion criteria

All shelter users present at that shelter on the day of interviewing were offered the chance to participate. Those that declined to participate or who were unable to give informed consent due to language difficulties or intoxication were excluded from the study. Double enrolment was avoided as participants’ names and dates of birth were known to interviewers. No incentive was offered for participation.

### Sample size calculation and statistical analysis

It was calculated that 150 participants would need to be chosen to provide a 95% confidence interval of ±8% for the prevalence of chronic pain. Data were entered into a spreadsheet and statistical analysis performed with GraphPad Prism version 4.0b (GraphPad Software, CA). Continuous data are expressed as the mean, with range and 95% confidence intervals (95% CI) where appropriate and non-continuous data are expressed as the median with the interquartile range (IQR). Groups were compared using non-parametric statistical tests: the Komolgorov-Smirnov test was used to examine data for normal distribution; where data were not distributed normally, the Mann-Whitney test was used. Relationships between continuous data were examined using linear regression, the gradient of the best-fit slope with 95% CI and r ^2^ values are reported. Statistical significance was indicated by a p value of less than 0.05.

### Institutional approval

The study was approved by the University of Cambridge Psychology research ethics committee (approval references 2009.73 and 2011.41).

## Results

One hundred and sixty-eight homeless people were invited to participate in the study: 150 (89.3%) completed the interviews, but 14 declined to participate, two were too intoxicated and two did not speak English. Of those completing the study, 134 (89.3%) were male and 16 (10.7%) were female; their mean age was 42.2 years (range 23–73 years, 95% CI 40.1–44.5 years).

Original data collected from interviews with homeless participants The original data collected from the Brief Pain Inventory (BPI), Short Form McGill (SF-McGill) and Leeds Assessment of Neuropathic Symptoms and Signs (LANSS) questionnaires. There is also data on the intake of prescribed and unprescribed medications, alcohol consumption, and the reasons for homelessness. This questionnaire was designed by the study team to capture demographic data that was not covered by the Brief Pain Inventory (BPI), Short Form McGill Pain (SF-McGill) or Leeds Assessment of Neuropathic Symptoms and Signs (LANSS) questionnaires used in the study.

### Characteristics of homelessness including causes and duration

Fifty-one participants (34.0%) reported sleeping rough on the streets, 95 (63.3%) used night shelters, and four (2.7%) were sleeping with friends or in squats. The mean duration of homelessness was 58.5 months (range 0.1–384.0 months, 95% CI 50.4–66.5 months).

The most common cause of homelessness in the 111 participants who were asked was breakdown of family relationships, cited by 44 (39.6%) participants. Further causes were: financial difficulties (29.6%); drug or alcohol misuse (17.4%); leaving prison or the armed services (11.9%); and health problems (10.9%). Twelve participants (8%) reported multiple causes of homelessness; hence the sum of percentages exceeds 100%.

### Prevalence, duration and intensity of pain

Of the 150 shelter users interviewed, 107 reported experiencing pain in the past 24 hours (71.3%, 95% CI 67.5%–74.8%). The reported mean duration of pain experienced was 78.3 months (range 0.1–480.0 months, 95% CI 66.4–90.2); 89 of 107 (83.2%) had experienced pain for more than three months suggesting an overall prevalence of 59.3% (95% CI 55.1%–63.0%). The worst, least and average pain intensities experienced are summarised in Table 1.

**Table 1.** The severity of pain experienced by participants in the previous 24 hours, on a numerical rating scale from 0 to 10.

	Homeless people reporting pain (n=107)		
Pain intensity	Mean	Range	95% CI
Worst pain reported in past 24 h	7.2	2–10	6.8–7.7
Least pain reported in past 24 h	2.8	0–10	2.3–3.3
Average pain	5.1	0–10	4.6–5.5

### Location of pain

**Figure 1.**
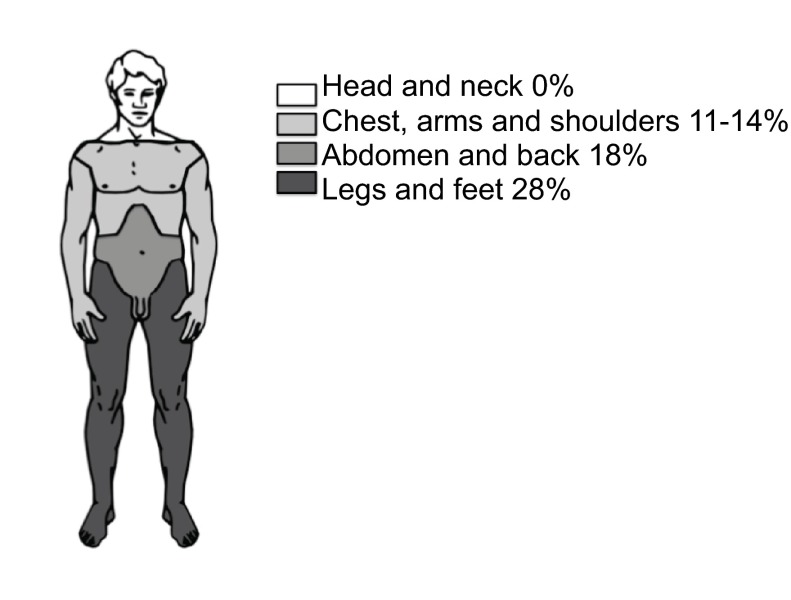
The distribution of pain reported by homeless shelter users.

The lower limbs were the most common site of pain, with 51.4% of participants reporting pain in this area ( Figure 1). Further areas affected were: abdomen, pelvis or back (36.9%); chest, arms and shoulders (25.2%); and head or neck (15.3%). Thirty-one participants (27.9%) reported more than one affected area; hence the sum of percentages exceeds 100%.

### Impact of pain on activities of daily living

General activity was the domain most affected by pain, with the majority (85.6%) of individuals with pain reporting that this aspect of their life was affected, the mean extent of this interference being 5.6 on an NRS of 0–10 ( Table 2). Relationships appeared to be the activity least affected by pain, with just over half (54.1%) of participants stating that their pain had an impact on relationships, and an average NRS score of 3.5. There was no statistical difference between the average pain experienced by those who slept outdoors and those who slept under a roof (p = 0.114).

**Table 2.** The activities of participants’ daily living affected by chronic pain, and the extent of perceived interference.

Activity	Number of participants in chronic pain who stated that pain had some effect on this activity (n)	Percentage of participants in chronic pain who stated that pain had some effect on this activity (%)	Mean NRS score for this activity
General activity	95	85.6%	5.6
Walking	83	74.7%	5.2
Mood	82	73.8%	5.2
Sleep	79	71.1%	5.2
Normal work	76	68.5%	4.7
Enjoyment of life	76	68.5%	4.6
Relationships	60	54.1%	3.5

### Influence of drugs and alcohol on pain

Fifty-nine of the 107 participants reporting chronic pain (55.1%) were taking over-the-counter and prescribed analgesics. Paracetamol (acetaminophen) was most popular, followed by non-steroidal anti-inflammatory drugs and opioids such as codeine, dihydrocodeine and morphine. Polypharmacy was common: 25 of the 59 participants (42.4%) taking over-the-counter and prescribed analgesics took more than one agent. None of the participants who reported no pain took over-the-counter or prescribed analgesics. Only four participants reported taking anti-neuropathic drugs: two were taking gabapentin, one dosulepin, and one amitriptyline. Other therapies were also used: three participants had access to physiotherapy; and a handful used complementary techniques such as acupuncture, osteopathy, and aromatherapy.

**Figure 2.**
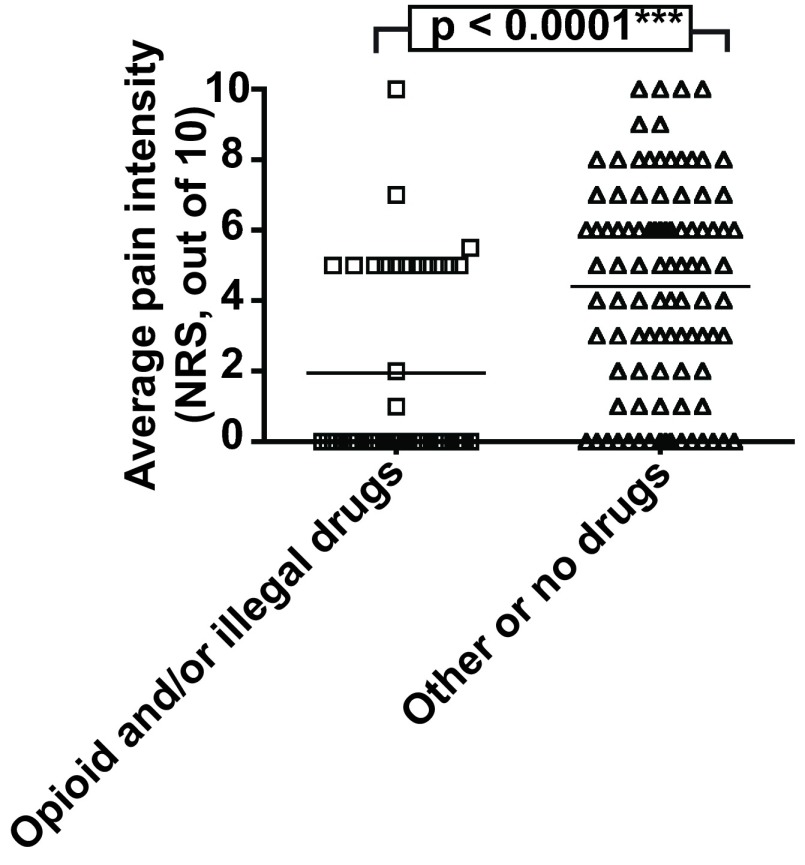
Pain intensity reported by homeless people categorised by drug use. Scatter plot to show the severity of average pain reported by homeless people who either take prescribed opioids, non-prescribed opioids, or cannabis; or those who take over the counter non-opioid analgesics or no drugs. Bars indicate means. Data compared using Mann-Whitney test.

Thirty-three participants (22.1%) admitted to using illegal drugs such as heroin, methadone, crack cocaine, cannabis, and non-prescribed diazepam; one participant declined to answer these questions. One participant reporting severe pain used cannabis for analgesia, and two who reported no pain said they did so because it was adequately treated by illegal methadone, heroin or diazepam. For those taking opioid analgesia, the mean reduction in pain intensity with their chosen regime was 46.3% (range 0%–100%, 95% CI 38.9%–53.8%). The average pain reported by those taking opioids, whether prescribed or unprescribed, was significantly lower than those who did not (p < 0.0001, Figure 2).

**Figure 3.**
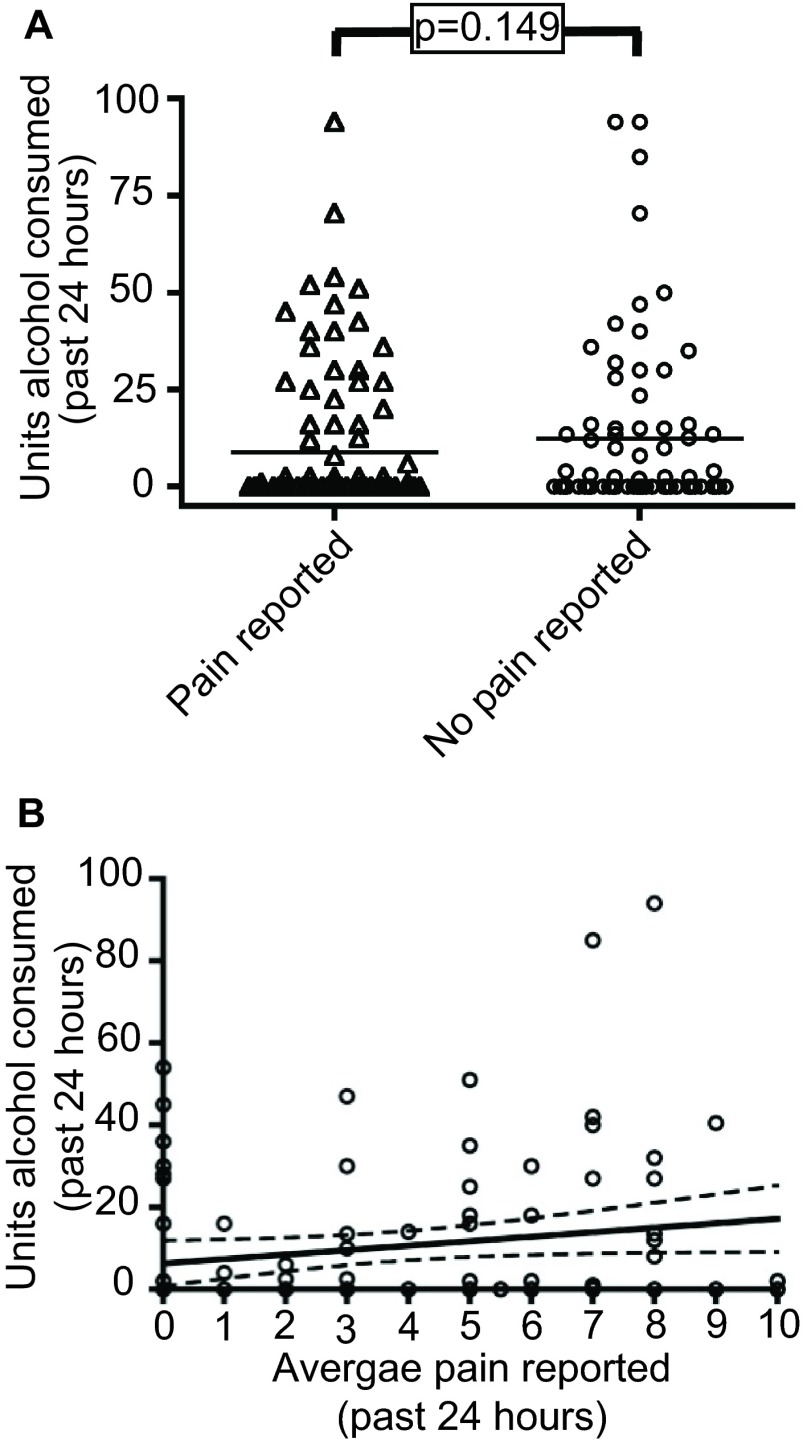
Pain intensity reported by homeless people categorised by alcohol use. **A**) Scatter plot showing the amount of alcohol consumed by homeless people reporting *versus* those who did not. Bars indicate means. **B**) Linear regression plot showing lack of relationship between quantity of alcohol consumed and intensity of pain reported. The unbroken line represents the best-fit slope and the broken lines the 95% confidence intervals (best-fit slope 0.579, 95% CI -0.06–2.24, r ^2^=0.034).

The majority (59.3%) of the 150 participants had not consumed alcohol in the previous 24 hours. Of the 61 (40.7%) who did, the mean number of units consumed was 19.1 (range 1.0–94.0, 95% CI 17.3–21.1). One participant declined to answer questions about alcohol. There was no apparent relationship between alcohol consumption and pain intensity: the number of units consumed by those reporting pain in the previous 24 hours was not significantly different (mean consumption 9.0 units *versus* 12.0 units, p = 0.149), nor was there a relationship between the quantity of alcohol consumed and pain experienced ( Figure 3).

### The prevalence of neuropathic pain

Of the 107 participants who reported pain, 90 completed the SF-MPQ questionnaire but only 59 completed the LANSS, as fewer participants wished to undergo the limited physical examination required completing this questionnaire. The median sensory pain score of SF-MPQ was 10 (IQR 5–16), the median affective pain score was 4 (IQR 0–7), and the median total combined component score was 14 (IQR 7–23). Table 3 shows a comparison of our data with that reported in clinical trials that used the SF-MPQ to assess the analgesic effect of anti-convulsant drugs in neuropathic pain ^29– 31^. Data shown from these studies are the mean scores of baseline data from the patients with neuropathic pain in the placebo arm of the trials. For ease of comparison this table contains our mean data, although arguably SF-MPQ is not a continuous scale. Fewer participants completed the LANSS scale: the median score was 6 (IQR 1–12); ten of those who did (16.1%) reported a score of 12 or more, highly suggestive of neuropathic pain ^26^. Therefore the LANSS data suggest a prevalence of neuropathic pain of 16.9%, and the SF-MPQ suggest that it is of a greater intensity than volunteers diagnosed with neuropathic pain entering clinical trials.

**Table 3.** Comparison of mean short-form McGill pain questionnaire (SF-MPQ) scores given by homeless participants with those given by volunteers with neuropathic pain enrolled in studies examining anti-neuropathic drugs.

	Study			Homeless group
	Backonja *et al* ^29^	Rice *et al* ^30^	Rowbotham *et al* ^31^	
Total score	21.0	17.1	18.7	14.5
Mean sensory score	Not given	13.2	14.5	10.7
Mean affective score	Not given	3.9	4.1	3.8

## Discussion

Pain is a substantial problem in homeless shelter users: 71.3% reported acute pain, and 59.3% fulfilled the criteria for chronic pain, the mean duration of which exceeded 6 years. The prevalence of chronic pain in our participants is substantially higher than that reported in several large population studies ^16– 18^. It also appears that a substantial component of the pain experienced is neuropathic in nature: 16.9% of participants who completed the LANSS scored 12 or more, which compares unfavourably with 8% in the general population ^32^.

It is difficult to ascertain whether pain is a potential cause or effect of homelessness in these individuals. Many factors associated with homelessness predispose to pain, and homeless people experience numerous barriers to health care, such as psychiatric illness, substance misuse, and the lack of a fixed address for correspondence ^33, 34^.

Lower limb pain was the most common location for pain in our study population. This is likely to be a result of lifestyle factors, such as ill-fitting shoes, long periods of standing, poor foot care, or sensory neuropathy. Many respondents commented that a lack of accessible facilities during the day resulted in a great deal of time spent on their feet. Abdominal pain was also common, which could result from opioid-induced constipation or alcohol-related diseases such as chronic pancreatitis, gastritis and peptic ulceration. The extent of the pain that shelter users experience exerts a considerable impact on their daily activities, with general activity and walking most affected. The severity of the pain reported by those with neuropathic symptoms is comparable to volunteers diagnosed with neuropathic pain enrolled into the placebo arms of three recent large trials of anti-neuropathic drugs ^29– 31^. The pain is also poorly controlled: just over half of those with chronic pain were using analgesia but the mean reduction in pain was only 46.3%, which compares unfavourably with the primary outcome measure of many trials of analgesic drugs, namely a 50% reduction in pain on a visual analogue scale ^35^. Inadequate pain control may result from reluctance to seek help with pain or investigation of underlying disease, for example due to a lack of awareness of help available, psychiatric illness, or low expectations of health professionals. Concerning treatment, physicians may be reluctant to prescribe opioids to individuals with a history of substance misuse, and non-steroidal anti-inflammatory drugs for those for those with a history of alcohol misuse ^19^.

Our findings corroborate previous studies that found a high prevalence of alcohol and substance misuse amongst homeless people, although it should be stressed that the majority of homeless people do not misuse alcohol or take illegal drugs ^4, 19^. We found no association between pain and alcohol use, but cannot exclude the possibilities that alcohol is being used to self-medicate, or that pain might arise as the result of alcohol-associated disease. Those using opioids, either prescribed, bought over the counter or obtained illegally, reported lower average pain intensity than those who did not. Many participants admitted obtaining opioids illegally specifically and solely for the purpose of pain relief. A small number also used illegally obtained cannabis and benzodiazepines for analgesia.

Our study has strengths and limitations. The time that our interviewers spent in the shelters and with homeless people meant they built up a rapport with a large number of homeless people, which is reflected in the high response rate. We found our participants pleasant, engaging and coherent; most defied our preconceived ideas about homeless people and did not fulfil the usual stereotypes. All interviewers were medical students, a particular advantage as participants reported speaking more freely and truthfully than perhaps they would with doctors. Previously, difficulties have been reported when measuring pain in the context of homelessness, perhaps due to participants’ lack of literacy skills ^21^. We chose questionnaires that are widely accepted as being valid in adults and read them through with our participants. We did not experience substantial problems with vague answers or engagement, and do not believe that introducing and validating new pain questionnaires specifically for homeless people is worthwhile as long as this paradigm is used. We reduced bias as far as possible by including a range of shelters and rough sleeping outreach services in our capital and provincial cities. Rough sleepers are some of the most difficult homeless people to engage, but a large proportion of our participants regularly slept outdoors. Therefore, we believe that our study population is as representative of the homeless population as practicable, and our findings could be generalised to other developed countries.

Most of the limitations of our study result from our desire not to overburden our participants with too many questionnaires and potentially intrusive personal questions. For example, we did not perform diagnostic interviews to collect data on psychiatric and physical disorders. The pain questionnaires used are independent of diagnosis and pathophysiology; they serve merely to quantify the amount of pain that a person is experiencing. This somewhat limits our ability to comment on causation of pain, and as some potential participants were intoxicated the possibility of inaccurate self-reporting of drug and alcohol use must also be considered. Although we collected high quality data from a relatively large number of homeless people, our study did not have sufficient power to detect differences between men and women, different age groups, or those with different social or economic reasons for homelessness.

Existing literature about pain among homeless persons is very limited, and a formal prevalence rate has not previously been reported. We found that almost two thirds of homeless people that we interviewed experienced chronic pain. Meeting the medical needs of homeless people is extremely difficult, so treating the underlying cause of the pain might not be possible. Our findings should raise awareness amongst healthcare professionals that chronic pain is common in homeless people and is generally inadequately controlled, and therefore requires thorough investigation and treatment. The drugs that our participants took to alleviate pain, either prescribed or unprescribed, afforded pain relief of less than 50%. Further studies are needed to establish the causes of pain in homeless persons, but it is notable that a large proportion reported symptoms and signs suggestive of chronic neuropathic pain. The observation that very few took anti-neuropathic drugs suggests that there may be opportunities to treat pain in this challenging but vulnerable population more effectively.
