# Supplementary material for: The nature and prevalence of chronic pain in homeless persons: an observational study
Source: F1000Res. 2013 Jul 30;2:164. [Version 1] doi: 10.12688/f1000research.2-164.v1 (PMC3886796; doi:10.12688/f1000research.2-164.v1)
Supplement: Original data collected from interviews with homeless participants — The original data collected from the Brief Pain Inventory (BPI), Short Form McGill (SF-McGill) and Leeds Assessment of Neuropathic Symptoms and Signs (LANSS) questionnaires. There is also data on the intake of prescribed and unprescribed medications, alcohol consumption, and the reasons for homelessness. This questionnaire was designed by the study team to capture demographic data that was not covered by the Brief Pain Inventory (BPI), Short Form McGill Pain (SF-McGill) or Leeds Assessment of Neuropathic Symptoms and Signs (LANSS) questionnaires used in the study. [file f1000research-2-870-s0000.tgz › Questionnaire.pdf]

Chronic Pain in the Homeless Additional Questions

UNIVERSITY OF CAMBRIDGE

All answers given are confidential and will only be seen by the research team.

1. Date of birth:

2. How long have you had the pain discussed in the questionnaires?

3. Are you currently taking any prescribed or unprescribed medications? If so what are these?

4. Approximately how much alcohol, if any, have you consumed in the last 24 hours?

5. Are you currently using any recreational drugs – if so which and how much?

6. How long have you been homeless/when did you first use homeless shelters?

7. What do you feel was the main contributing factor for becoming homeless?

- Relationship breakdown [ ]
- Family problems [ ]
- Drug and alcohol problems [ ]
- Health problems [ ]
- Financial [ ]
- Leaving prison [ ]
- Other [ ]

8. Where would you say you spend the majority of nights over the last weeks/months?

- Shelter [ ]
- Rough sleeping/streets [ ]
- Friend/relative's accommodation [ ]
- Other [ ]
