# Supplementary material for: The nature and prevalence of chronic pain in homeless persons: an observational study
Source: F1000Res. 2013 Jul 30;2:164. [Version 1] doi: 10.12688/f1000research.2-164.v1 (PMC3886796; doi:10.12688/f1000research.2-164.v1)
Supplement: Peer review history

# Peer review: Referee response for version 1

**Published:** 2013-10-25

**Authors:** Lara Weinstein

**Affiliations:** 1 Department of Family and Community Medicine, Jefferson Medical College, Thomas Jefferson University, Philadelphia , PA, USA

This is an interesting and well researched article on an important and little studied topic. I have a few comments for the authors to consider:

1. I would find it helpful to include a brief description of the questionnaires used in the study, perhaps with a comment on their reliability and validity.
2. In the discussion about lower limb pain you may want to mention trauma as a likely source in this population.
3. The relatively low percentage of women in the sample limits the generalisability to women.

I have read this submission. I believe that I have an appropriate level of expertise to confirm that it is of an acceptable scientific standard.

# Response

**Published:** 2013-10-28

**Authors:** Rebecca Fisher

**Affiliations:** Department of Primary Health Care Sciences, University of Oxford, UK

1. The questionnaires used in the study are well validated and were chosen by the study team in part for ease of use with our study population, and also to provide comprehensive coverage of both neuropathic and non-neuropathic pain. There is little work on how to best measure pain in the context of homelessness (see reference 21, Matter *et al*), and we felt a combination of these questionnaires would allow a comprehensive assessment of pain, that was not overly burdensome to participants. References 22-28 provide further information on the questionnaires used in this study.
2. We agree that trauma could have been added as a cause of lower limb pain in this population. Indeed several participants anecdotally mentioned abdominal stab wounds as contributing to their pain, and this could have been discussed further.
3. We agree that the low percentage of women in the study limits its generalisability. In our study the small number of female participants reflected the overwhelmingly male occupancy of the night shelters at which participants were recruited. It would be interesting to expand the study to shelters catering only for women. 

# Peer review: Referee response for version 1

**Published:** 2013-10-04

**Authors:** Benjamin Henwood

**Affiliations:** 1 School of Social Work, University of Southern California, Los Angeles, CA, USA

This is a well written and well conceived study that focuses on an important topic: the nature and prevalence of pain among individuals experiencing homelessness. This is an obvious and overlooked area of research that represents an important target of clinical intervention. The methods are clear, appropriate, and appear well executed. The findings confirm what one might expect: there are high rates of chronic pain among the homeless. They also add insight into the type of pain individuals experience including high rates of neuropathic pain. The notion that these individuals use drugs to self-medicate the effects of pain seems only partially supported, suggesting that pain is an important consideration, but only one of many in terms of understanding substance use in homeless populations. High rates of the use of acetaminophen raise questions about potential liver damage and toxicity, and it would be worth considering homeless individuals' knowledge on this topic, which is perhaps a point that could be raised in the discussion. One final suggestion is to consider whether and how providing housing would help address or alleviate pain among this population.

I have read this submission. I believe that I have an appropriate level of expertise to confirm that it is of an acceptable scientific standard.
